# Nephrogenic Diabetes Insipidus following an Off-Label Administration of Sevoflurane for Prolonged Sedation in a COVID-19 Patient and Possible Influence on Aquaporin-2 Renal Expression

**DOI:** 10.1155/2022/3312306

**Published:** 2022-03-11

**Authors:** Camie Dupuis, Arnaud Robert, Ludovic Gerard, Johann Morelle, Pierre-François Laterre, Philippe Hantson

**Affiliations:** ^1^Department of Intensive Care, Cliniques Universitaires St-Luc, Université Catholique de Louvain, Brussels, Belgium; ^2^Division of Nephrology, Cliniques Universitaires St-Luc, Université Catholique de Louvain, Brussels, Belgium

## Abstract

During the recent COVID-19 pandemic, the rapidly progressive shortage of intravenous sedative drugs led numerous intensive care units to look for potential alternatives in patients requiring mechanical ventilation for severe acute respiratory distress syndrome (ARDS). Inhalational sedation using the AnaConDa® device for sevoflurane administration is a possible option. In a 54-year-old COVID-19 patient with severe ARDS requiring extracorporeal membranous oxygenation (ECMO), sevoflurane on AnaConDa® device was administered for 8 days but was complicated by the development of nephrogenic diabetes insipidus (NDI). Other causes of NDI or central diabetes insipidus were reasonably excluded, as in other previously published cases of NDI in ICU patients receiving prolonged sevoflurane-based sedation. In addition, the postmortem examination suggested a lower expression of aquaporin-2 in renal tubules. This observation should prompt further investigations to elucidate the role of aquaporin-2 in sevoflurane-related NDI. Inhaled isoflurane sedation is a possible alternative.

## 1. Introduction

During the recent COVID-19 pandemic, numerous patients with acute respiratory distress syndrome (ARDS) in the intensive care unit (ICU) required prolonged mechanical ventilation with deep sedation and neuromuscular blocking agents in order to be adapted to ventilator settings. Worldwide, it appeared, however, rapidly that the risk of drug shortages might arise. Particularly, the delivery of intravenous sedative agents became a major problem for many hospital pharmacies [1]. In this view, alternative ways of sedation had to be explored and among them, the use of volatile anesthetic agents. Mainly adapted for general anesthesia purpose in the operating room, they are becoming increasingly popular in ICUs worldwide since the development of gas reflectors, such as AnaConDa® (SEDANA Medical, Uppsala, Sweden), which allow their easy administration with an open-circuit ICU ventilator. Among them, sevoflurane has many potential pharmacokinetic and pharmacodynamic advantages for critically ill patients [2]. The efficacy of sevoflurane sedation has been documented in some ICU trials, but the analysis of safety issues is limited by the number of patients included and by the absence of comparison with alternative techniques [3–6]. In particular, the occurrence of nephrogenic diabetes insipidus (NDI) has only been recently documented in a small number of ICU patients after prolonged inhalational sedation with sevoflurane [7, 8]. This renal injury appears different from the nephrotoxicity described historically with methoxyflurane. We describe a recent case of NDI with postmortem investigation for aquaporin-2 expression in renal tubules.

## 2. Case Description

**Figure 1:**
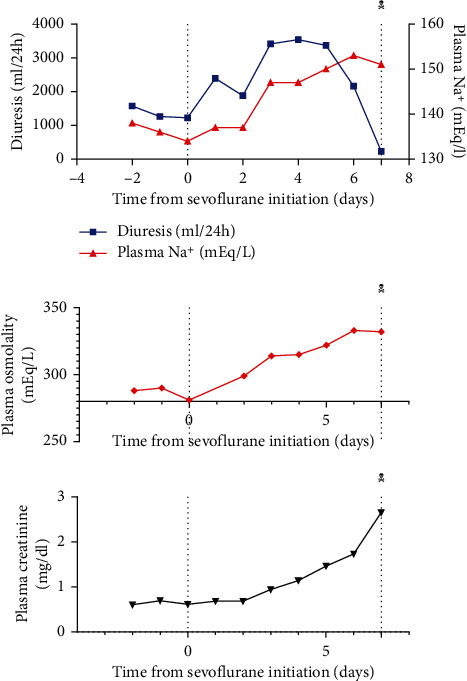
Laboratory investigations.

**Figure 2:**
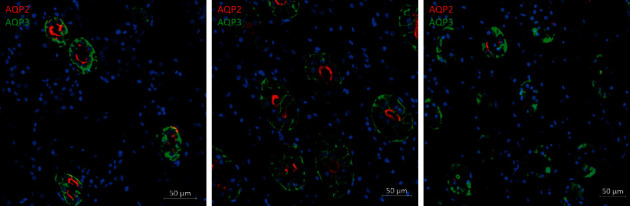
Representative pictures of double immunostaining with anti-aquaporin-3 (AQP3; green channel) and anti-aquaporin-2 (AQP2; red channel) antibodies in kidney sections from a control patient (left), a patient with severe COVID-19 (middle), and a patient with severe COVID-19 and sevoflurane-associated nephrogenic diabetes insipidus (right). Nuclei are stained with DAPI (4′,6-diamidino-2-phenylindole).

A 54-year-old man (75 kg body weight, 180 cm height) was admitted to the intensive care unit (ICU) (April 2020, second wave of epidemic) with severe COVID-19 related ARDS. His previous medical history was unremarkable, he never smoked and did not report alcohol consumption. The patient was not vaccinated for SARS-CoV2. Symptoms started one week before, with fever, dry cough, and dyspnea after exertion. Chest X-ray examination was consistent with diffuse COVID-19-related pneumonia, and infection was confirmed by RT-PCR. The patient presented marked hypoxemia with PaO_2_/FiO_2_ ratio 103 under high-flux nasal oxygen therapy (0.60 FiO_2_). Less than 24 hours after ICU admission, orotracheal intubation was required for mechanical ventilation with FiO_2_ 1.0. The initial sedative drug regimen included propofol, midazolam, ketamine, and sufentanil, in addition to neuromuscular blockage with cisatracurium. A transient improvement was noted after the change for the prone position on mechanical ventilation. Unfortunately, the patient developed on day 6, ventilator-acquired pneumonia with *Pseudomonas aeruginosa.* This resulted in a worsening of gas exchanges despite several adaptations of ventilator settings and introduction of inhaled nitric oxide (NO). It was then decided to start with veno-venous extracorporeal membranous oxygenation (ECMO) by the same day. Sedation was then provided by intravenous propofol (4 mg/kg/h), ketamine (0.8 mg/kg/h), clonidine (0.5 mcg/kg/h), sufentanil (5 mcg/h), and midazolam (0.15 mg/kg/h). On day 8, due to the relative shortage of intravenous sedatives and neuromuscular blocking agents in our hospital, sedation based on inhaled sevoflurane administration with AnaConDa® (Sedana Medical, Danderyd, Sweden) device was introduced and then continued for 8 days. Among intravenous sedatives, propofol, sufentanil, and clonidine were withdrawn, while ketamine (0.5 mg/kg/h) and midazolam (0.15 mg/kg/h) were continued. Cisatracurium administration was also stopped. Sevoflurane end-tidal concentration of 1% vol and injection rate of 6–10 ml/h were used to achieve a score of −2 to 0 on the Richmond Agitation Sedation Scale (RASS). The evolution of plasma osmolality, sodium, creatinine, and urine output is displayed in Figure 1. Fluid administration was increased to compensate for excess fluid losses and a therapeutic challenge with IV desmopressin (4 mcg) was performed but did not influence urine output. The diagnosis of nephrogenic diabetes insipidus (NDI) was suspected. Other causes of NDI were carefully excluded, and the review of medications suggested the role of sevoflurane. Unfortunately, the patient developed refractory vasoplegic shock and died on day 15 (while still under sevoflurane sedation) with a drop in urine output and rapid development of acute renal failure. The shock was likely related to the recent infection as it appeared that *P. aeruginosa* was resistant to the initial antimicrobial therapy. The late evolution of renal function was also more likely related to shock rather than to drug toxicity. A postmortem examination was obtained. A double immunostaining for aquaporin-2 (AQP2-Sigma A7310) and aquaporin-3 (AQP3-Sigma HPA014924) was performed on formaldehyde-fixed paraffin-embedded sections of the kidney from the patient using a sequential staining protocol, described previously [9] (Figure 2). The same staining protocol was applied to sections of the kidney of a control patient and of a patient with severe COVID-19 who had not received sevoflurane. Kidney sections were analyzed under a Zeiss LSM800 confocal microscope (Carl Zeiss). Visual analysis of the kidney sections suggested a decreased expression of AQP2 in our patient, as compared to the two control patients, suggesting a selective loss of AQP2, related to sevoflurane infusion. Conversely, the expression of AQP3 seemed unchanged. However, autolysis precluded extensive quantification of AQP2 and AQP3 expression.

## 3. Discussion

Despite its extensive use in operating rooms, and, more recently, in ICUs, only a few cases of polyuria or NDI associated with sevoflurane have been described [7, 8, 10–13]. It has to be remembered that the use of conventional ICU ventilators coupled to specific devices (such as AnaConDa®) for prolonged sevoflurane administration is still off-label. In addition, robust data on long-term use of volatile anesthetics are missing [14]. The exact incidence of sevoflurane-related NDI is not precisely known. In a recent retrospective analysis on 35 ICU brain-injured patients who had received sevoflurane with the AnaConDa® device, seven presented with NDI during their ICU stay. There was clearly an influence of the duration of exposure and the end-tidal concentrations of sevoflurane [13]. Polyuria stopped after a median time of 43.5 h after sevoflurane discontinuation. There was also a significant trend in an increase in serum creatinine at the end of sevoflurane administration but none of the patients developed chronic kidney injury. More specifically, NDI was recently reported in two COVID-19 patients who had received sevoflurane sedation for a respective duration of 8 and 9 days [15]. Sevoflurane was administered using ventilators of the operating theater along with continuous end-tidal gas monitoring.

The exact mechanism of sevoflurane-related NDI is not well elucidated. It seems that sevoflurane could temporarily reduce renal concentrating ability [7, 8]. Besides, the link between sevoflurane and renal concentrating ability is probably related to aquaporin-2 (AQP2), arginine-vasopressin (AVP)-regulated water channel localized in renal collecting duct cells, and involved in the regulation of water permeability. Morita et al. recently showed that sevoflurane-induced a transient and reversible impairment of urine concentrating capabilities through a reduced AQP2 expression, despite increased AVP concentrations during general anesthesia [12]. It has been previously documented that patients with central diabetes insipidus had an increase in urinary aquaporin-2 excretion in response to the administration of vasopressin, while patients with NDI did not [16]. In our patient, the relative decrease in the expression of AQP2 in the kidney sections, compared to controls, suggested that sevoflurane-induced NDI is indeed probably related to the loss of AQP2. To our knowledge, this is the first time that the expression of AQP 2 is assessed on kidney sections of a patient undergoing prolonged sevoflurane anesthesia.

On the other hand, nephrotoxicity related to volatile anesthetics has been linked to the production of inorganic fluoride (IF) through hepatic cytochrome P450-mediated metabolism [17, 18]. However, two clinical studies failed to retrieve a relationship between high IF levels and renal impairment, in particular with the use of sevoflurane [19, 20].

Finally, SARS-CoV2 infection itself can cause a specific dysfunction of the kidney proximal tubule with a pattern mimicking renal Fanconi syndrome [9]. However, there is no direct evidence that it could induce NDI nor affect aquaporin-2 renal expression.

There are some limitations to this single observation. Urine osmolality was not measured, but the patient failed to show any improvement of polyuria under desmopressin, strongly suggesting NDI. The patient was also not explored by a serum arginine-vasopressin (AVP) determination nor with a brain magnetic resonance imaging (MRI), but a central origin for diabetes insipidus appeared extremely unlikely. Finally, we were not able to quantify precisely AQP2 expression on kidney sections, due to some degree of postmortem autolysis. To our best knowledge, no other study has previously explored renal aquaporin-2 expression in relationship with sevoflurane-induced NDI and further investigations are required. Since September 2021, Sedaconda® (inhaled isoflurane) has been approved in a number of European countries for sedation of mechanically ventilated adult patients during intensive care. Specific follow-up of unusual renal effects would be of interest.

## Additional Points

A specific report was sent to the regulatory authorities of the country.
